# Mineral Cements

**Published:** 1895-04

**Authors:** S. J. Willey


ARTICLE IV.

### MINERAL CEMENTS.

BY PROF. S. J. WILLEY.

For a long while there have been on the market mineral cements, phosphate of zinc, insoluble cements, diamond cements, and a great number whose titles would seem to indicate that the substance to be put into the form of a plastic filling is not excelled even by the diamond itself. All of these cements, subjected to the ordinary chemical analysis, disclose the fact that they are made of similar substances, and their real differences consist largely in the method of preparing them.

It is not uncommon for dentists to be informed that oxid of zinc, put into a crucible properly luted, and the whole placed in an ordinary fire and allowed to remain for an hour or two, constitutes a cement of very excellent quality; and that they may purchase at any drug shop dilute phosphoric acid, and the two will make a cement to fill cavities with safety both to the patient and their own reputation. Experiments with these things sometimes meet with tolerable success; but there cannot be by this kitchen method that uniformity of production which emanates from a scientific conception of the uses to which plastic fillings are put. To have uniform results, materials must be prepared scientifically.

The glacial phosphoric acid that is useful for the ordinary purpose of the druggist will not necessarily be useful for the dentist, because the needs of the two are different. What methods are to be employed in the dehydration of aqueous phosphoric acid. The specific gravity and heat intensely belong solely to the functions of him who knows what these things should be, to make a proper chemical combination with his powder. That one may know the degrees of his Baume and of his Fahrenheit, and also of the fact that aqueous vapor should be eliminated from the phosphoric acid, when chemical combination has been properly brought about by the insertion of other substances into it, are important; but it is still more important, both scientifically and commercially, to know how this material can be uniformly produced and its integrity maintained.

The methods necessary to produce this liquid in a form which will give the best results do not come within the profitable purview of him who uses this fluid. They are too costly.

A test employed by some manufacturers to determine when the fluid is properly prepared is to affirm that the fluid should be of the consistency of glycerin, as though all glycerin is of the same consistency, regardless of the influence of quality or heat and cold. Just as we can never determine whether a substance is alkaline or acid except by tests by the proper agents, so you can not tell by sight alone when the fluid is in proper condicion; scientific apparatus alone must determine it. In this, as in some other matters, sight fails.

The same criticisms which hold in relation to liquids for cements will hold largely in reference to the powder. Should it be an oxid of zinc? If so, why should not the raw oxid of zinc answer as well? Should it be calcined? Why? Does the principle of dehydration enter here the same as in the fluid; and, if so, what intensity of heat must be employed to produce the dehydration of the powder? Is it less an oxid after calcining than it was before? Does it possess the same hydroscopic qualities before as after? These should concern the manufacturer, and he must, by actual test in manipulation, and integrity both in and out of the mouth, determine what must control him in the preparation of the powder for cements.

While the powder and the fluid have been prepared according to known and scientific rules, there is nothing left then, if these two are chemical substances, but to bring them together under proper conditions, and then we shall have a new compound instead of phosphoric acid or the zinc; we may likely have a zinc phosphate that enters the tooth cavity and performs the functions of which the dentist very well knows its utility. When these two substances are brought together chemical action is the result, heat is evolved and a new substance formed; therefore the more intimate the fluid and the powder become, the slower will be the setting of the cement, the finer the grain, and the more solid the filling.

Every molecule of the powder should come in contact with every molecule of the fluid. The rules given for mixing are the result of this principle.

The mineral cements The Wilmington Company offer the profession have been prepared according to the lines here laid down. These cements cannot be mixed like a hod-man mixes his mortar, because the chemical combinations will not form in the same way, nor will the proper results be obtained from a physical point of view. Therefore, we have deemed it wise to give the following suggestions to guide the dentist in mixing these mineral cements whose utility and value are becoming more and more apparent as they are used more and more by the profession.

Only a very little practice is required to become an adept in mixing the material, so that the mass will be exactly of that plastic character best suited to the cavity to be filled. A failure in mixing will always result by using an excess of powder. Cleanliness is very important. The glass slab to be used should be thoroughly cleaned, and the spatula smooth and tolerably stiff.

While it is very difficult to formulate any exact set of rules which will meet the requirements of every dentist, because it is difficult to find any two who are governed by the same rules; yet, after a long series of experiments and consultations with the very best operators in the profession, we find that the following rule will be an excellent guide to those who have not yet adopted the methods here suggested:

Pour on a small glass plate enough fluid for your purpose, put beside it enough cement powder for the filling. Draw the powder gradually into the fluid, and with a stiff spatula quickly rub the mixture into the consistency of cream, bearing hard all the while on the spatula, gradually manipulating it without any more powder. When the mass has become sufficiently plastic, apply it to the tooth cavity in the usual manner.

It is necessary to emphasize the fact that no more powder must be added to the mass when it has been worked to the consistency of cream, and that continued manipulations, without the addition of any more powder, will gradually bring it to that degree of plasticity which is essential to a hard, unshrinkable, and bony mass.—*Items of Interest.*

